# Characterization of a family IV esterase from extremely halophilic archaeon *Haloarcula japonica*

**DOI:** 10.1007/s00792-024-01370-2

**Published:** 2024-12-03

**Authors:** Hiromichi Kato, Shota Ambai, Fumiya Ikeda, Koji Abe, Satoshi Nakamura, Rie Yatsunami

**Affiliations:** School of Life Science and Technology, Institute of Science Tokyo, 4259 Nagatsuta, Midori-ku, Yokohama, 226-8501 Japan

**Keywords:** Esterase, Family IV, Hormone-sensitive lipase family, Halophilic esterase, Extremely halophilic archaeon, *Haloarcula japonica*

## Abstract

The novel esterase gene *lipP1*, which encodes HjEstP1, was discovered in the genome of the extremely halophilic archaeon *Haloarcula japonica*. A homology search and sequence alignment revealed that HjEstP1 is a member of family IV esterases with conserved GXSXG and HGGG motifs. *lipP1* was expressed in its parental strain, and recombinant HjEstP1 was purified and characterized. Optimal pH and temperature of HjEstP1 were 6.0 and > 60 °C, respectively. HjEstP1 showed higher activity with increasing NaCl concentration, and optimal NaCl concentration was > 4.5 M. Furthermore, HjEstP1 preferentially hydrolyzed *p*NP and glycerol esters with short chain fatty acids. To our knowledge, this is the first report of an esterase from an extremely halophilic archaeon obtained via homologous expression.

**Supplementary Information:**

The online version contains supplementary material available at 10.1007/s00792-024-01370-2.

## Introduction

Esterases (carboxylesterases, EC 3.1.1.1) and lipases (triacylglycerol lipases, EC 3.1.1.3) are carboxylic ester hydrolases that catalyze the cleavage and formation of ester bonds (Bornscheuer 2002). Both enzymes share a typical $\alpha /\beta$ hydrolase fold with a catalytic triad consisting of Ser, Asp (Glu), and His (Ollis et al. 1992). Esterases and lipases possess similar sequences and structures but are distinguished in terms of the acyl chain length specificity of their substrates (Reyes-Duarte et al. 2005). For example, esterases prefer triacylglycerols with acyl chains shorter than C_6_, whereas lipases preferentially hydrolyze long-chain triacylglycerols (Bornscheuer 2002).

Microbial esterases and lipases were originally categorized into eight families based on their amino acid sequences and biochemical properties (Arpigny and Jaeger 1999). As the discovery of novel enzymes, the classification has been expanded to 35 families and 11 true lipase subfamilies to date (Hitch and Clavel 2019). Among them, family IV, also referred to as the hormone-sensitive lipase (HSL) family, has some conserved sequence motifs such as the GXSXG (amino acid one-letter code; Xs are any amino acid) motif, including the catalytic Ser, and the HGGG motif containing oxyanion hole residues (Hemilä et al. 1994; De Simone et al. 2000). Some of Family IV esterases characterized to date show thermal stability (Byun et al. 2007; Li et al. 2015; Miguel-Ruano et al. 2021), solvent tolerance (Park et al. 2006; Gricajeva et al. 2019; Lu and Daniel 2021), and activity toward tertiary alcohol esters (Henke et al. 2002; Bassegoda et al. 2013; Ngo et al. 2013; Yan et al. 2014). Family IV esterases with such properties have great potential for various industrial applications, such as additives in detergents and foods, bioremediation, and biotransformation (Kim 2017).

Enzymes from extremely halophilic archaea (haloarchaea) can function under high salt conditions with low water content and are expected to be used in aqueous/organic and nonaqueous media (Sellek and Chaudhuri 1999). Thus, haloarchaeal esterases are important biocatalysts for ester production and further exploration of novel enzymes is required to promote their applications. In 2009, a haloarchaeal esterase Hm EST from *Haloarcula marismortui* was purified and characterized by heterologous expression in *Escherichia coli* (Müller-Santos et al. 2009). As haloarchaeal enzymes are frequently produced as inclusion bodies in *E*. *coli*, homologous expression at high salt concentration is desirable (Martínez-Espinosa 2020). However, homologous expression of a haloarchaeal esterase has not yet been reported.

*Haloarcula japonica* is an extremely halophilic archaeon with predominantly triangular disc-shaped cells (Takashina et al. 1990). This organism shows optimal growth at 2.6–4.3 M NaCl in the presence of 160 mM MgSO_4_ and produces large amount of cell surface glycoproteins (CSG) (Nakamura et al. 1992; Nishiyama et al. 1992). Employing the powerful *csg* promoter, several recombinant proteins from extremely halophilic archaea were successfully produced in *H*. *japonica* (Yatsunami et al. 2010; Onodera et al. 2013; Sueda et al. 2021).

In this study, the gene encoding a putative family IV esterase was identified and cloned from the genome of *H*. *japonica*. The esterase gene was expressed in *H*. *japonica*, and the recombinant esterase was purified and characterized.

## Materials and methods

### Strains, media, and plasmids

Strains JM109 and JM110 of *E. coli* were used as hosts for gene cloning and unmethylated plasmid preparation, respectively. Unmethylated plasmids have been used to transform *H*. *japonica* and improve the transformation efficiency by avoiding restriction barriers (Holmes et al. 1991). *E*. *coli* strains were cultured at 37 °C in LB medium, and 50 μg/mL of ampicillin was added to the medium if necessary.

*H*. *japonica* strain TR-1 (JCM 7785), which was a donor of genomic DNA, was also used as a host for gene expression. *H*. *japonica* was cultured at 37 °C in a complex medium containing 20% NaCl and 4% MgSO_4_・7H_2_O (DasSarma and Fleischmann 1995). Pravastatin (8 μg/mL; provided by Daiichi-Sankyo Co., Ltd.) was used instead of mevinolin.

pWL102 is an *E*. *coli*-haloarchaea shuttle vector (Lam and Doolittle 1989) and its derivative pWL102-P_*csg*_ contains the *H*. *japonica csg* promoter upstream of the *Nde* I/*Bam*H I sites (Nagaoka et al. 2021).

### Sequence analyses

The amino acid sequence was analyzed using the ProtParam program (https://web.expasy.org/protparam/) provided by Expasy (Gasteiger et al. 2005). Signal peptides were searched using SignalP-5.0 (https://services.healthtech.dtu.dk/services/SignalP-5.0/) (Almagro Armenteros et al. 2019). Homology search was conducted through the BLAST program (http://blast.ncbi.nlm.nih.gov/Blast.cgi) using Protein Data Bank database and Protein–Protein BLAST algorithm. Multiple sequence alignment was performed using Clustal Omega (https://www.ebi.ac.uk/Tools/msa/clustalo/) (Madeira et al. 2022).

The nucleotide sequence of *lipP1* has been deposited in the DNA Data Bank of Japan (DDBJ) under accession number LC762521.

### Gene cloning, expression, and purification of the recombinant protein

Genomic DNA was extracted from *H*. *japonica* as previously described (DasSarma and Fleischmann 1995). *lipP1* was amplified from the genome via PCR with following primers; lipP1( +) (5’-GTACATATGCGAGCCCAAGAACCAC-3’) containing an additional *Nde* I site, and lipP1(-) (5’-GGATCCTAGTGGTGGTGGTGGTGGTGTTTCAGTGCAGACTCGAGG-3’) containing an additional 6 × His-tag sequence and *Bam*H I site. The PCR product was then inserted into the *Sma* I site of pUC119. The resulting plasmid was digested with *Nde* I and *Bam*H I and then ligated into the *Nde* I/*Bam*H I sites of pWL102-P_*csg*_ to construct a haloarchaeal expression plasmid for HjEstP1, pJLipP1.

Recombinant HjEstP1 was obtained according to the previous report (Nagaoka et al. 2021). Namely, unmethylated pJLipP1 obtained from *E*. *coli* strain JM110 was introduced into *H*. *japonica* as previously described (Onodera et al. 2013). The transformant was cultured to express *lipP1*, and cells were harvested by centrifugation (4,400 × g, 4 °C, 20 min). The cells were suspended in the standard buffer [3.0 M NaCl containing 20 mM NaH_2_PO_4_-NaOH (pH 7.5)], and the suspension was sonicated to disrupt the cell. After ultracentrifugation (100,000 × g, 4 °C, 1 h), supernatant was collected to obtain a cytoplasmic fraction. The cytoplasmic fraction was dialyzed against the standard buffer containing 20 mM imidazole, and applied on a Ni^2+^-immobilized HiTrap Chelating HP (GE Healthcare, Illinois) column. Proteins absorbed on the column were eluted using a linear gradient of imidazole (20–500 mM). The eluted fraction was dialyzed against the standard buffer without imidazole to obtain a purified HjEstP1 solution. Protein purity was confirmed via SDS-PAGE using a 12.5% polyacrylamide separating gel (Laemmli 1970) with an SDS-PAGE Molecular Weight Standards Low Range (Bio-Rad, CA) as a marker. Protein concentration was determined following the Lowry method (Lowry et al. 1951) using a DC protein assay kit (Bio-Rad) with bovine serum albumin (BSA) as a standard.

### Standard enzyme assay

The esterase activity toward *p*-nitrophenyl (*p*NP) propionate (C_3_; FUJIFILM Wako Pure Chemical, Osaka, Japan) was quantitatively determined spectrophotometrically by measuring the amount of liberated *p*NP. Before the enzymatic reaction, purified HjEstP1 was suitably diluted using standard buffer in the presence of 0.1% BSA. The addition of BSA prevented a decrease in HjEstP1 activity during dilution (data not shown). For the standard assay, a substrate solution was prepared as follows: 20 mM *p*NP propionate (C_3_) solution (dissolved in acetonitrile) was gently mixed with 50 mM Britton-Robinson buffer (pH 7.0) containing 3.05 M NaCl at a ratio of 1:48 (v/v). After 784 µL of substrate solution was pre-incubated at 37 °C for 2 min, reaction was initiated by adding 16 µL enzyme preparation. The absorbance at 405 nm was continuously recorded at 37 °C (physiological temperature of *H*. *japonica*) for 1 min using a UV-1650 spectrophotometer (Shimadzu, Kyoto, Japan). The reaction temperature was regulated using a CPS-240 controller (Shimadzu). Background hydrolysis, which was measured in the same manner using 0.1% BSA-containing standard buffer instead of enzyme preparation, was subtracted. The initial velocity was calculated from the linear portion of the absorbance change over time using the molar extinction coefficient of *p*NP (ε = 1.2 × 10^4^ M⁻^1^ cm⁻^1^). One unit of enzyme activity was defined as the amount of enzyme liberating 1 µmol of *p*NP per min.

### Effects of pH, temperature, and NaCl concentration on enzyme activity

The effect of pH was determined at 37 °C in the presence of 3.0 M NaCl and 2.0% acetonitrile. The reaction was performed following the standard assay procedure with 50 mM Britton-Robinson buffer adjusted to each pHs. Britton-Robinson buffer is known to show buffering capacity over a wide pH range (Britton and Robinson 1931). The absorbance was recorded at 348 nm, which is the isosbestic wavelength of* p*NP and *p*NP ions (Manco et al. 1998). At this wavelength, the absorbance of *p*NP is not influenced by pH, maintaining similar sensitivity among the different pH values. The enzyme activity was calculated using the molar extinction coefficient of *p*NP at 348 nm (ε = 5.2 × 10^3^ M⁻^1^ cm⁻^1^). The effects of temperature and NaCl concentration were determined at pH 7.0 in the presence of 3.0 M NaCl and 2.0% acetonitrile, and at 37 °C and pH 7.0 in the presence of 2.0% acetonitrile, respectively. The reaction was performed following the standard assay procedure at different temperatures and NaCl concentrations, and the enzyme activity was calculated using the molar extinction coefficient of *p*NP at 405 nm under each reaction condition.

### HjEstP1 activity toward various substrates

HjEstP1 activity toward substrates with different acyl chain lengths was investigated using *p*NP acetate (C_2_; Nacalai Tesque, Kyoto, Japan), *p*NP propionate (C_3_), *p*NP butyrate (C_4_; Nacalai Tesque), *p*NP hexanoate (C_6_; Tokyo Chemical Industry, Tokyo, Japan), and *p*NP octanoate (C_8_; Sigma-Aldrich, MO, USA). In this assay, the substrate solution was vigorously mixed for 1 min to solubilize as much substrate as possible. The enzymatic reaction was performed according to the standard assay procedure.

The esterase activity toward glycerol esters was qualitatively assessed using agar plates emulsified with glycerol esters. 50 mM Britton-Robinson buffer (pH 7.0) containing 3.05 M NaCl and 1.5% agar was autoclaved. Before the solution temperature dropped below 70 °C, 1.0% (v/v) tripropionin (Tokyo Chemical Industry) or olive oil (Nisshin OilliO Group, Tokyo, Japan) was added and vigorously mixed to form an emulsion, and then poured into disposable Petri dishes to prepare agar plates. Wells of approximately 8 mm in diameter were created to be poured enzyme solution. The agar plates were incubated at 37 °C, and hydrolytic activity was evaluated by halo formations.

## Results and discussion

### Sequence analyses of *lipP1*

The complete genome sequence of *H*. *japonica* was determined in 2007, comprising two chromosomes (chromosomes 1 and 2) and three plasmids (pHJ1, 2, and 3) (Nakamura et al. 2011). On the plasmid pHJ1 of *H*. *japonica*, an esterase/lipase homolog *lipP1* was discovered. *lipP1* is 939 bp long and encodes a protein consisting of 312 amino acids with a molecular mass of 34,060 Da. According to SignalP-5.0, the deduced amino acid sequence of *lipP1* did not include a signal peptide, suggesting that *lipP1* encodes an intracellular protein. Moreover, the *lipP1* product exhibits a high acidic amino acids (Asp + Glu) ratio, implying that it could display halophilic properties (Lanyi 1974; Fukuchi et al. 2003; Graziano and Merlino 2014).

A BLASTp search revealed that the deduced amino acid sequence of *lipP1* showed homology with family IV esterases such as Est8 from a metagenome (Pereira et al. 2017), PestE from *Pyrobaculum calidifontis* (Hotta et al. 2002), and Est2 from *Alicyclobacillus acidocaldarius* (Manco et al. 1998) (Table 1). On the other hand, the *lipP1* product shares only 40% sequences with its homologs, indicating that this is a novel esterase. Therefore, the protein encoded by *lipP1* was named HjEstP1.

**Table 1:** Comparisons of sequence identity, query cover, and acidic amino acids contents among HjEstP1 homologs

Esterase	Origin	Identity [%]	Query cover* [%]	Acidic amino acids (Asp + Glu) ratio [%]
HjEstP1	*Haloarcula japonica*	100.0	100	19.9
Est8	Metagenome	45.1	93	12.5
PestE	*Pyrobaculum calidifontis*	46.6	84	11.2
EST2	*Alicyclobacillus acidocaldarius*	41.6	97	13.2
Sto-Est	*Sulfurisphaera tokodaii* (*Sulfolobus tokodaii*)	38.5	97	11.2
AFEST	*Archaeoglobus fulgidus*	43.7	89	14.8
Hm EST	*Haloarcula marismortui*	39.8	30	17.2

*Percentage of query sequence aligned

**Fig. 1:**
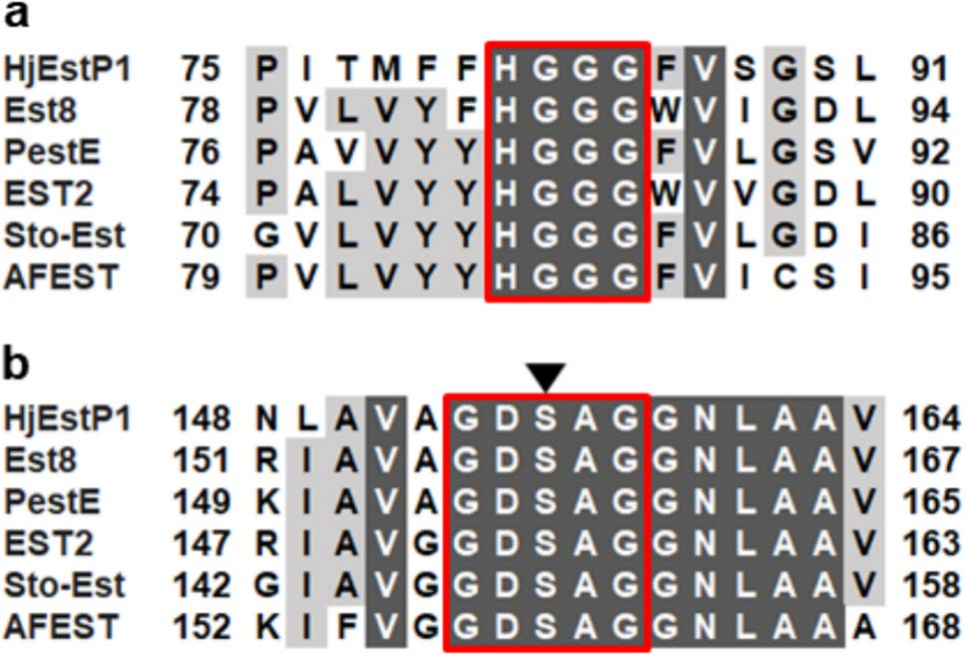
Partial amino acid sequence alignment of HjEstP1 and its homologs. **a** Region surrounding the HGGG motif. **b** Region surrounding the GXSXG motif. HGGG and GXSXG motifs were boxed in red lines. Conserved residues among all sequences were indicated using white letters on a black background. Highly conserved residues were highlighted in grey. Catalytic Ser within GXSXG motif was indicated by an arrowhead

Based on multiple sequence alignment, HjEstP1 contained the GXSXG and HGGG motifs, both of which are conserved among family IV esterases (Fig. 1). The GXSXG motif contains the catalytic serine and can be seen in other lipase and esterase family as well. The HGGG motif could be involved in stabilizing the reaction intermediate by forming an oxyanion hole. The catalytic triad was estimated to consist of Ser156, Glu251, and His281. One of these, Ser156, was confirmed to be a catalytic residue, as HjEstP1 lost its activity upon substitution of Ser156 with Ala (Figs. S1 and S2).

### Overexpression of *lipP1* in *H. japonica* and purification of recombinant HjEstP1

**Fig. 2:**
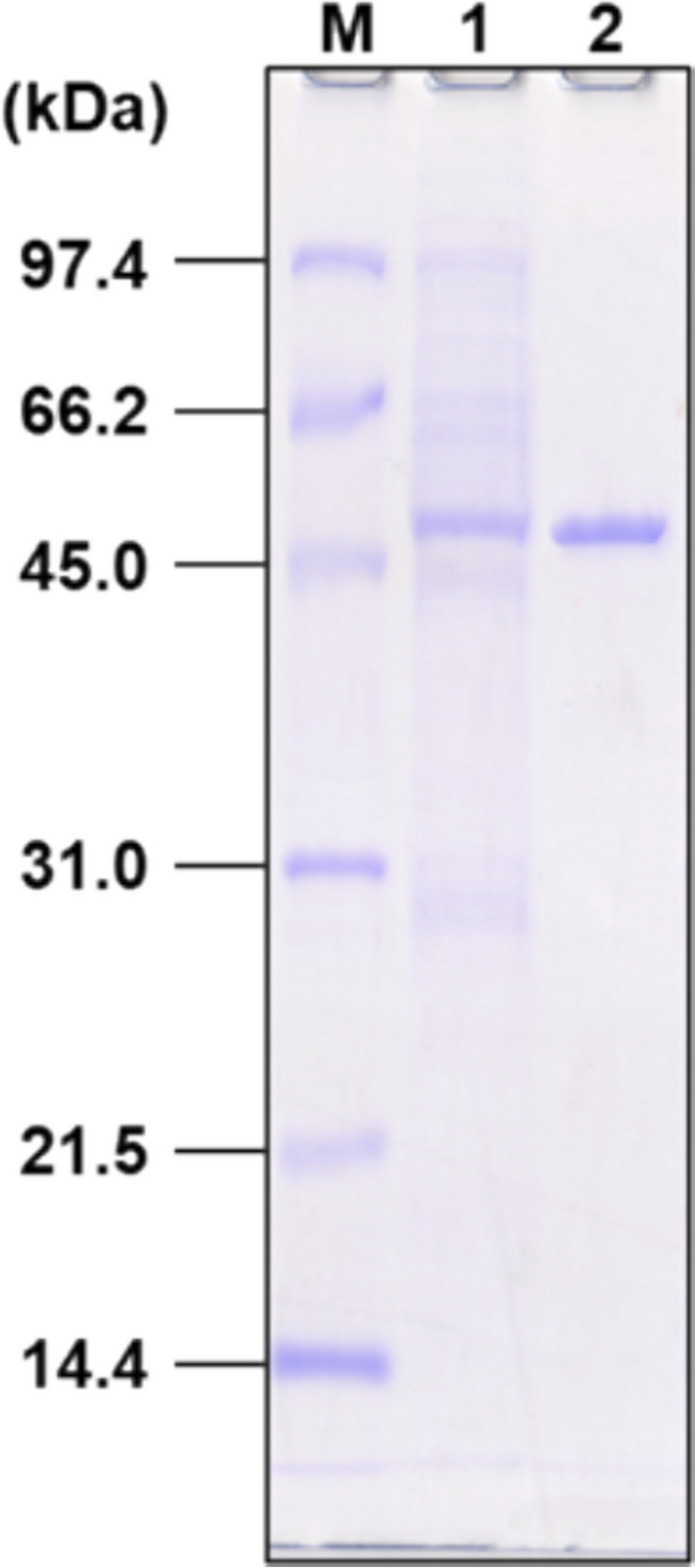
SDS-PAGE of recombinant HjEstP1. Lane M, markers; lane 1, cytoplasmic fraction; lane 2, purified HjEstP1

The *lipP1*-containing DNA fragment was PCR-amplified from the *H*. *japonica* genome and cloned to construct the expression plasmid pJLipP1. In pJLipP1, the *csg* promoter and C-terminal His-tag sequences were located upstream and downstream of *lipP1*, respectively. *H*. *japonica* harboring pJLipP1 was cultured, and recombinant HjEstP1 was purified from the cytoplasmic fraction using affinity chromatography in the presence of 3.0 M NaCl. Purified HjEstP1 produced a single band on SDS-PAGE, with a molecular mass of approximately 50 kDa, although the theoretically calculated value was approximately 35 kDa (including His-tag, Fig. 2). Haloarchaeal proteins contain many acidic amino acids, which make it difficult for SDS to bind to them. Therefore, the molecular masses of haloarchaeal proteins are often overestimated in SDS-PAGE (Izotova et al. 1983; Wakai et al. 1997). Purified HjEstP1 showed esterase activity toward *p*NP propionate (C_3_) with a specific activity of 1,044 U/mg under standard assay conditions (pH 7.0, 37 °C, 3.0 M NaCl). To the best of our knowledge, this is the first report of the homologous expression of a haloarchaeal esterase.

### Effects of pH, temperature, and NaCl concentration on the activity of HjEstP1

**Fig. 3:**
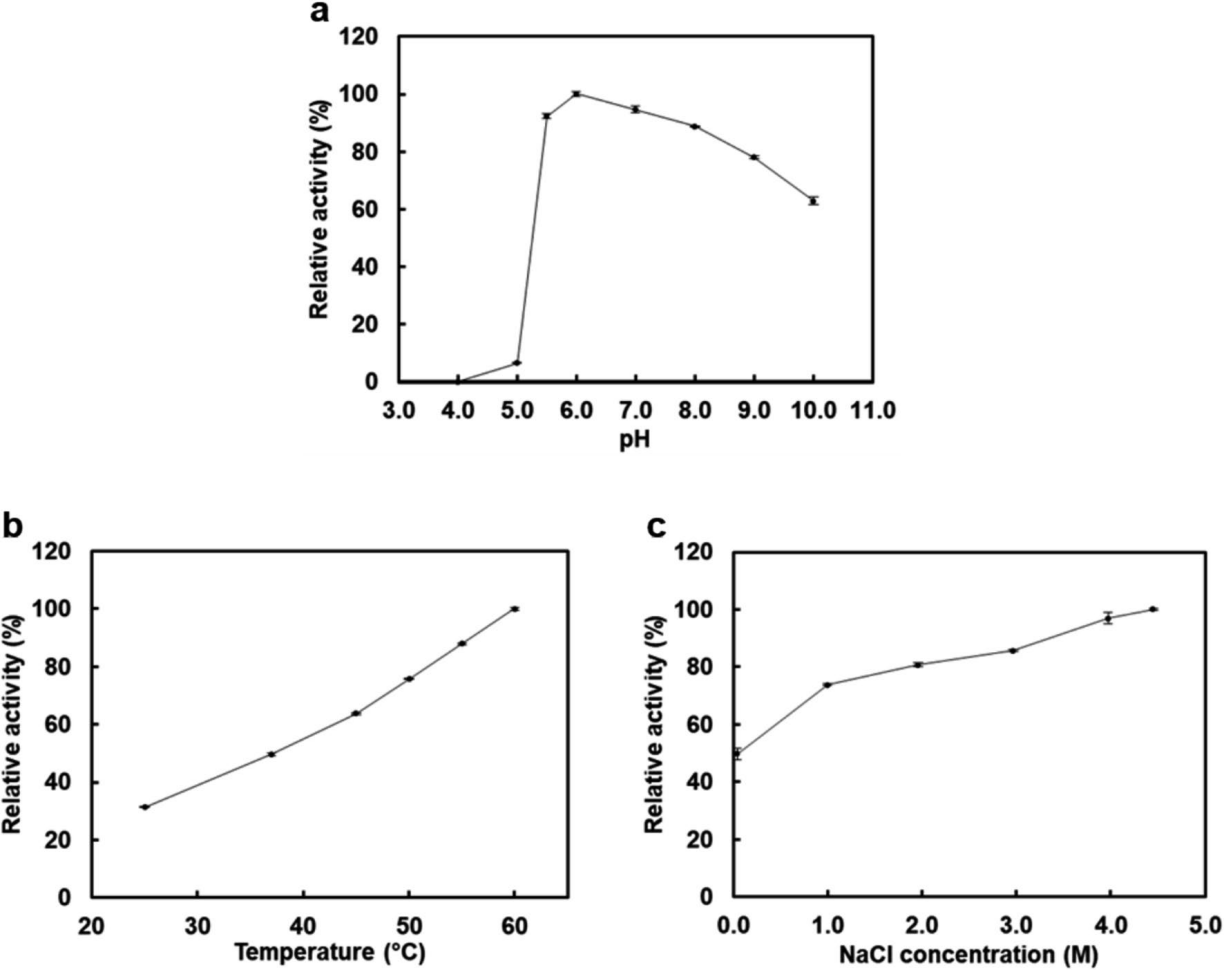
Effects of pH (**a**), temperature (**b**), and NaCl concentration (**c**) on the activity of HjEstP1. **a** The effect of pH (37 °C, 3.0 M NaCl). **b** The effect of temperature (pH 7.0, 3.0 M NaCl). **c** The effect of NaCl concentration (37 °C, pH 7.0). The reaction was performed using *p*NP propionate (C_3_) as substrate. The relative activity was defined as the percentage of maximum activity at pH 6.0 (**a**), 60 °C (**b**), and 4.5 M NaCl (**c**). Each measurement was performed in triplicate and data were shown as the mean ± SD

The effect of pH on enzyme activity was measured using different pH buffers. HjEstP1 showed relatively high activity at a broad pH range from 5.5 to 7.0, and the optimal pH was 6.0 (Fig. 3a). The optimal pH of HjEstP1 was slightly lower than that of other esterases (pH 7.0–9.0) (Manco et al. 1998, 2000; Hotta et al. 2002; Suzuki et al. 2004; Pereira et al. 2017). At pH 5.0, significant loss of activity was observed. In addition, the activity gradually decreased with increasing pH above 6.0, and finally, the activity decreased to 63% at pH 10.0. However, under alkaline conditions, the activity may be underestimated owing to rapid auto-hydrolysis of the substrate.

The effect of temperature on enzyme activity was determined under various temperatures ranging from 25–60 °C. The activity of HjEstP1 increased proportionally with increasing reaction temperature and the optimal temperature was likely higher than 60 °C (Fig. 3b). As the temperature controller equipped with the spectrophotometer is recommended to be used under 60 °C by the supplier, the activity could not be determined above 60 °C. Although the temperature profile of HjEstP1 appeared unusual considering the growth temperature of the host species, several recombinant proteins from *H. japonica* showed maximum activity at relatively high temperatures (Onodera et al. 2013; Sueda et al. 2021; Nagaoka et al. 2021).

The effect of NaCl concentration on enzyme activity was investigated. As the NaCl concentration increased, HjEstP1 showed higher activity, with maximum activity at > 4.5 M NaCl (Fig. 3c). At the NaCl concentration close to 0 M, the activity decreased to nearly half that at 4.5 M NaCl. HjEstP1 exhibited relatively high activity at more than 1.0 M NaCl, demonstrating the typical properties of halophilic enzymes that require high salt concentrations for activity. Similar to HjEstP1, culture supernatants from some halophilic archaea showed highest esterase activity at high NaCl concentrations such as 4.0 M or 4.5 M (Ozcan et al. 2009). On the other hand, many bacterial halotolerant esterases isolated from saline environment and metagenomic resources exhibited maximum activity at lower than 3.0 M (Jiang et al. 2012; Wang et al. 2013, 2020; Xin and Hui-Ying 2013; De Santi et al. 2016; Huo et al. 2017; Lu and Daniel 2021).

### HjEstP1 activity toward various substrates

**Fig. 4:**
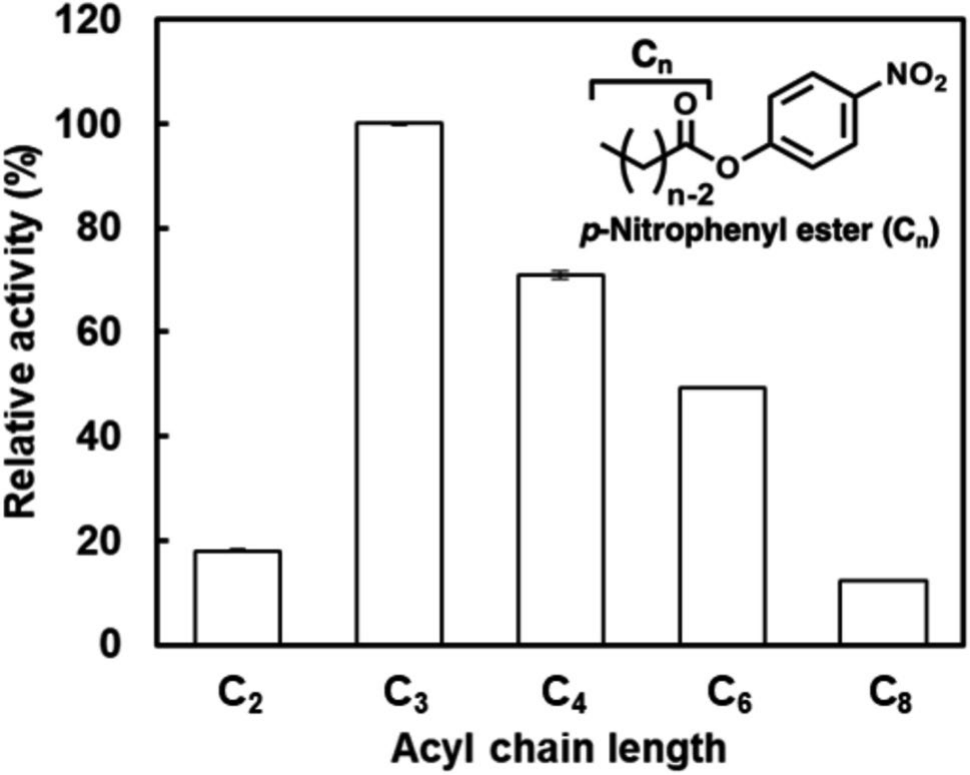
Acyl chain length specificity of HjEstP1 toward *p*NP ester. Reaction was conducted at pH 7.0 and 37 °C in the presence of 3.0 M NaCl and 2.0% acetonitrile. The relative activity was defined as the percentage of maximum activity toward *p*NP propionate (C_3_). Each measurement was performed in triplicate and data were shown as the mean ± SD

HjEstP1 activity was investigated using *p*NP esters with different acyl chain lengths (C_2_–C_8_). HjEstP1 hydrolyzed all the *p*NP esters examined and exhibited maximum activity toward* p*NP propionate (C_3_) (Fig. 4). It showed relatively high activity toward short-chain esters (less than C_6_), confirming that HjEstP1 is an esterase. In contrast, HjEstP1 had difficulty hydrolyzing *p*NP acetate (C_2_). The relative activity toward *p*NP acetate (C_2_) was approximately 20%, which was almost identical to that toward *p*NP octanoate (C_8_).

**Fig. 5:**
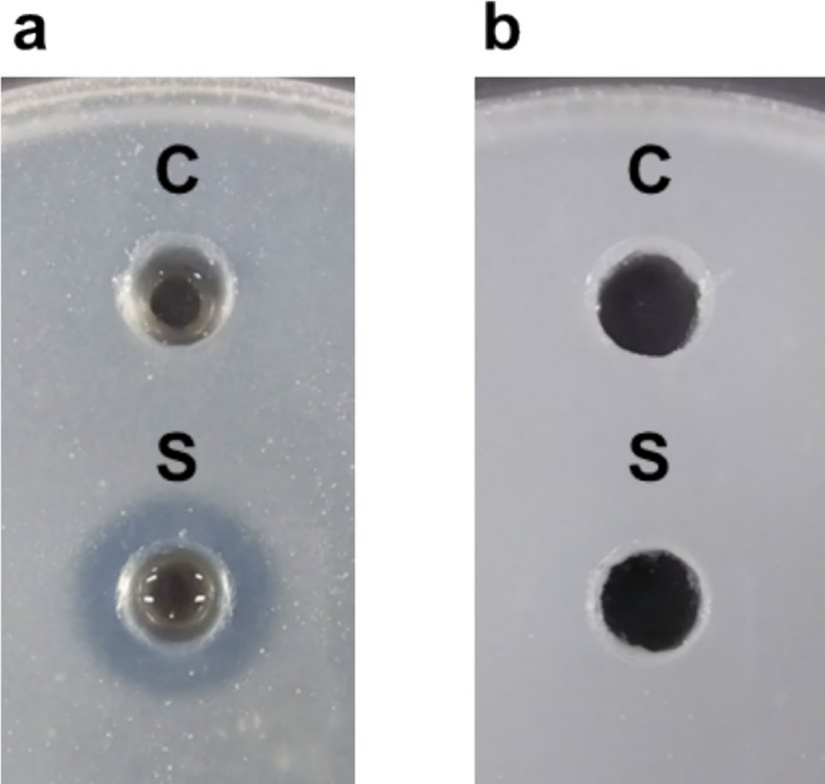
Agar plate hydrolytic assay of glycerol esters. **a** Tripropionin-containing agar plate after incubation for 4 h. **b** Olive oil-containing agar plate after incubation for 24 h. Reaction was conducted at pH 7.0 and 37 °C in the presence of 3.05 M NaCl. C, standard buffer (control); S, HjEstP1

To investigate the ability of HjEstP1 to hydrolyze glycerol esters, a qualitative assay was conducted using agar plates emulsified with glycerol esters. Emulsified agar plates appeared turbid; however, the turbidity diminished after the hydrolysis of glycerol esters. Therefore, the formation of a halo around a well with the enzyme solution indicates the hydrolysis of glycerol esters. In this assay, tripropionin (triacylglycerol with C_3_ acyl chains) and olive oil (mixed glycerol esters with acyl chains longer than C_16_) were used as substrates. On the tripropionin plate, a clear halo was observed around the well containing HjEstP1 (Fig. 5a). In contrast, a halo did not appear around the control well without the enzyme, confirming that halo formation was the result of enzymatic hydrolysis. In contrast to the tripropionin plate, no halo was observed on the olive oil plate even after 24 h of incubation (Fig. 5b). These results suggested that HjEstP1 hydrolyzes glycerol esters with relatively short acyl chains.

HjEstP1 hydrolyzes various esters such as* p*NP and glycerol esters, as long as the acyl chains are relatively short. This tendency of HjEstP1 is consistent with the characteristics of other family IV esterases (Kim 2017). Thus, HjEstP1 may probably have a small substrate-binding pocket covered with a cap structure, similar to other family IV esterases that are structurally well described (Manco et al. 2001; Yang et al. 2015; Pereira et al. 2017). X-ray crystallographic analysis is currently underway to clarify the relationship between chain length preference and the 3D structure of HjEstP1.

## Conclusion

In this study, *H*. *japonica lipP1*, which encodes HjEstP1, was cloned and expressed in its parental strain. For the first time, recombinant HjEstP1 was successfully obtained by homologous expression. The activity of HjEstP1 increased at high NaCl concentrations, demonstrating the typical properties of halophilic enzymes. Moreover, HjEstP1 preferentially hydrolyzed the short-chain substrates of *p*NP and glycerol esters.

## Supplementary Information

Below is the link to the electronic supplementary material.

Supplementary file1 (PDF 307 KB)

## Data Availability

Data will be made available on request.
