# Supplementary material for: Characterization of a family IV esterase from extremely halophilic archaeon Haloarcula japonica
Source: Extremophiles. 2024 Dec 3;29(1):7. doi: 10.1007/s00792-024-01370-2 (PMC11614938; doi:10.1007/s00792-024-01370-2)
Supplement: Supplementary file 1 — Supplementary file1 (PDF 307 KB) [file 792_2024_1370_MOESM1_ESM.pdf]

Supplementary Information

Extremophiles

Characterization of a family IV esterase from extremely halophilic archaeon *Haloarcula japonica*

Hiromichi Kato, Shota Ambai, Fumiya Ikeda, Koji Abe, Satoshi Nakamura, Rie Yatsunami*

School of Life Science and Technology, Institute of Science Tokyo, 4259 Nagatsuta, Midori-ku, Yokohama, 226-8501, Japan

*Corresponding author
E-mail address: yatsunami.r.dfe1@m.isct.ac.jp

Supplementary Method

A mutant substituted Ser156 by Ala (S156A) was prepared by site-directed mutagenesis. Modified *lipP1* fragment was amplified using pJLipP1 as template with following primers: S156A[F] (5’-GCGACGCCGCCGGTGGGAAC-3’) containing an Ala substitution, and Mega[R] (5’-CTGAACCTATGAACCCGGGTCGGACAACAAC-3’) containing a *Sma* I site. The obtained DNA fragments were utilized as mega-primers in the following PCR to construct whole plasmid. As a template, pJLipP1 from *E. coli* strain JM109 was used. After the PCR, the reaction mixture was treated with *Dpn* I and methylated template pJLipP1 was digested. To restore nicking, plasmids were introduced into *E. coli* strain JM109. Targeted plasmid was selected by *Sma* I digestion, and Ala substitution was confirmed by nucleotide sequencing. Purification of the recombinant S156A and activity measurement was performed as described in the body.

Supplementary Figures

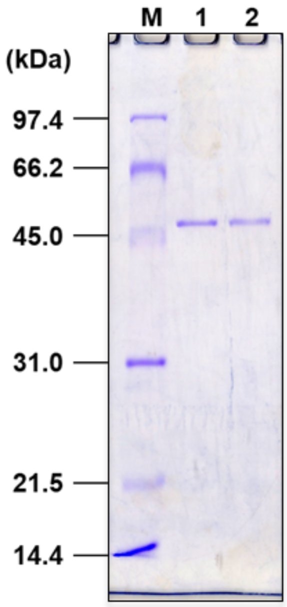

Fig. S1 SDS-PAGE of recombinant S156A

Lane M, markers; lane 1, purified HjEstP1; lane 2, purified S156A. Purified S156A showed a single band at almost the same location as purified HjEstP1.

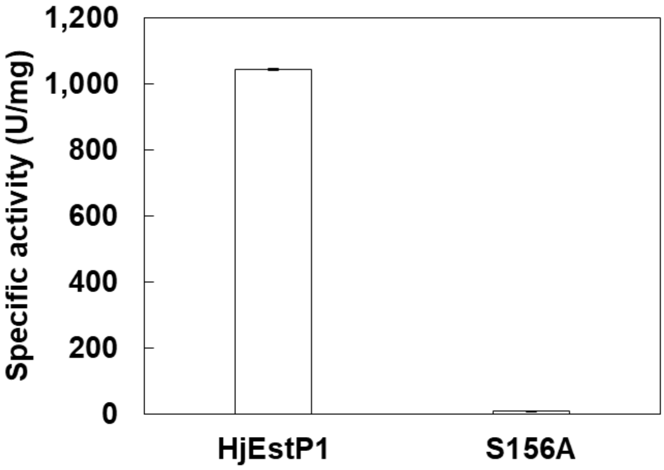

Fig. S2 Comparison of specific activity of HjEstP1 and S156A

Reaction was conducted at pH 7.0 and 37 °C in the presence of 3.0 M NaCl and 2.0% acetonitrile. *p*NP propionate (C_3) was used as substrate. Each measurement was performed in triplicate and data were shown as the mean ± SD.
